# Evidence of stress imprinting with population‐level differences in two moss species

**DOI:** 10.1002/ece3.5205

**Published:** 2019-05-09

**Authors:** Weiqiu Liu, Jianqu Xu, Wei Fu, Xiangyuan Wang, Chunyi Lei, Yunfeng Chen

**Affiliations:** ^1^ Guangdong Key Laboratory of Plant Resources, School of Life Sciences Sun Yat‐sen University Guangzhou China; ^2^ Department of Scientific Research and Education Heishiding Nature Reserve Zhaoqing China

**Keywords:** bryophytes, desiccation, osmolytes, phytohormones, population differentiation, stress imprint

## Abstract

Plants are often repeatedly exposed to stresses during their lives and have a mechanism called stress imprinting that provides “memories” of stresses they experience and increases their ability to cope with later stresses. To test hypotheses that primed bryophytes can preserve their stress imprinting after 6 days of recovery and induce higher levels of osmolytes and ROS‐scavenging activities upon later stress exposure, and there exist population‐level differentiation in their desiccation defenses, we transplanted samples of two populations of each of two moss species, *Hypnum plumaeforme* and *Pogonatum cirratum*, in a nature reserve in southern China. After 16 months of acclimation, sets of each population were subjected to control, one‐time desiccation stress, duplicated desiccation stress and cross‐stress (low temperature stress followed by desiccation stress) treatments. Levels of oxidant enzymes, osmolytes, and phytohormones in the samples were then determined. The desiccation stress generally led to increases in activities or contents of superoxide dismutase, guaiacol peroxidase, catalase, proline, soluble sugars, soluble proteins, and stress hormones including abscisic acid (ABA), jasmonates (JA), and salicylic acid (SA), with differences between both species and populations. After a 6‐day recovery period, contents of phytohormones (including ABA, JA, SA, and cytokinins) in stressed *H. plumaeforme* had substantially fallen toward control levels. The duplicated and cross‐stress treatments generally led to further accumulation of proline, soluble sugars, and soluble proteins, with further increases in activities of antioxidant enzymes in some cases. Furthermore, significant differences between allochthonous and native populations were found in contents of malondialdehyde and osmolytes, as well as antioxidant enzyme activities. Our results confirm the hypotheses and highlight the importance of osmolytes in mosses' stress responses.

## 1 INTRODUCTION

Land plants are frequently challenged by diverse environmental stresses as they cannot escape from adverse environments. Hence, they have evolved vital survival mechanisms that enable them to sense and adapt to the stresses. Phytohormones are key messengers that integrate external stress signals with internal metabolic activities (Kohli, Sreenivasulu, Lakshmanan, & Kumar, 2013; Peleg & Blumwald, 2011; Verma, Ravindran, & Kumar, 2016; Verslues, 2016). Abscisic acid (ABA), jasmonates (JA), salicylic acid (SA), and ethylene, which are defined as stress phytohormones, generally increase in stress conditions (Bruce, Matthes, Napier, & Pickett, 2007). Others, such as auxins, gibberellins, and cytokinins, are also involved in stress responses via complex signaling networks (Verma et al., 2016; Verslues, 2016). Phytohormones regulate stress responses that often include increases in antioxidant capacity and accumulation of osmolytes (Cvikrová et al., 2012; De Diego et al., 2013; Hüve, Bichele, Tobias, & Niinemets, 2006; Liu, Lei, Jin, et al., 2015; Nagao, Minami, Arakawa, Fujikawa, & Takezawa, 2005; Zhao, Shi, Liu, Jia, & Li, 2015). Furthermore, information about experienced stresses (“stress memories” or “stress imprints”) can be stored in plants and may often increase their tolerance of recurrent stresses (Bruce et al., 2007; Nikiforou & Manetas, 2018). Phytohormone regulation and accumulation of antioxidants and osmolytes in response to previous stress are considered important elements of stress imprinting (Asensi‐Fabado, Oliván, & Munné‐Bosch, 2013; Bruce et al., 2007; Walter, Jentsch, Beierkuhnlein, & Kreyling, 2013). As various abiotic stresses, such as low temperature and drought, can induce some similar physiological responses, they may cause cross‐stress imprinting in higher plants (Blödner, Skroppa, Johnsen, & Polle, 2005; Walter et al., 2013).

For bryophytes, plenty of studies has demonstrated that experienced desiccation can make them adapt to desiccation stress better (Beckett, 1999; Beckett, Marschall, & Laufer, 2005; Bopp & Werner, 1993; Dilks & Proctor, 1976; Schonbeck & Bewley, 1981a, 1981b; Werner, Ros Espín, Bopp, & Atzorn, 1991); however, this stress imprinting tends to wear off with the extension of the recovery time, and slower desiccation rates produce longer imprinting times (Brinda, Stark, Clark, & Greenwood, 2016). For naturally growing mosses in the field, water between dense individuals generally leads to relatively slow desiccation rates and the intervals between dry events varied. It has been reported that a period of more than 7 days is required to remove the acclimation experience (Hájek & Vicherová, 2014; Hellwege, Dietz, Volk, & Hartung, 1994; Stark, Greenwood, Brinda, & Oliver, 2014), and studies have also demonstrated that levels of osmolytes and antioxidants may increase in mosses upon exposure to low temperature or water stress, and may remain relatively high even after alleviation of stresses for 10 days (Liu et al., 2016; Liu, Lei, Jin, et al., 2015). So, we hypothesized that the priming information can be stored in mosses for a certain time (e.g., 6–10 days) and induce higher tolerance to a later stress. A complicating factor is that populations within a species' distribution are often exposed to different environmental conditions and thus develop local adaptations (Briggs, 1972; Chambers & Emery, 2016; Lázaro‐Nogal et al., 2016). As both selective environmental pressures and gene flow limitations drive differentiation, between‐population distances and dispersal potentials both affect the extent of population‐level differentiation (Chambers & Emery, 2016; Korpelainen, Pohjamo, & Laaka‐Lindberg, 2005). Moreover, due to the limited dispersal potential of bryophytes, significant population differentiation can be found within quite a small area (Briggs, 1972; Snäll, Ribeiro, & Rydin, 2003). Although population differentiation of bryophytes has received some attention, studies have generally focused on their morphological and life‐history traits (Hedderson & Longton, 2008) or direct determination of their genetic structure (Pohjamo, Korpelainen, & Kalinauskaitė, 2008; Wang, Zhu, & Wang, 2012). Between‐population differences in their physiological traits have received much less attention despite their importance in species' fitness. However, it is clearly important to characterize and distinguish such differentiation and stress imprinting mechanisms (if present) in attempts to clarify bryophytes' suites of stress responses.

**Figure 1:**
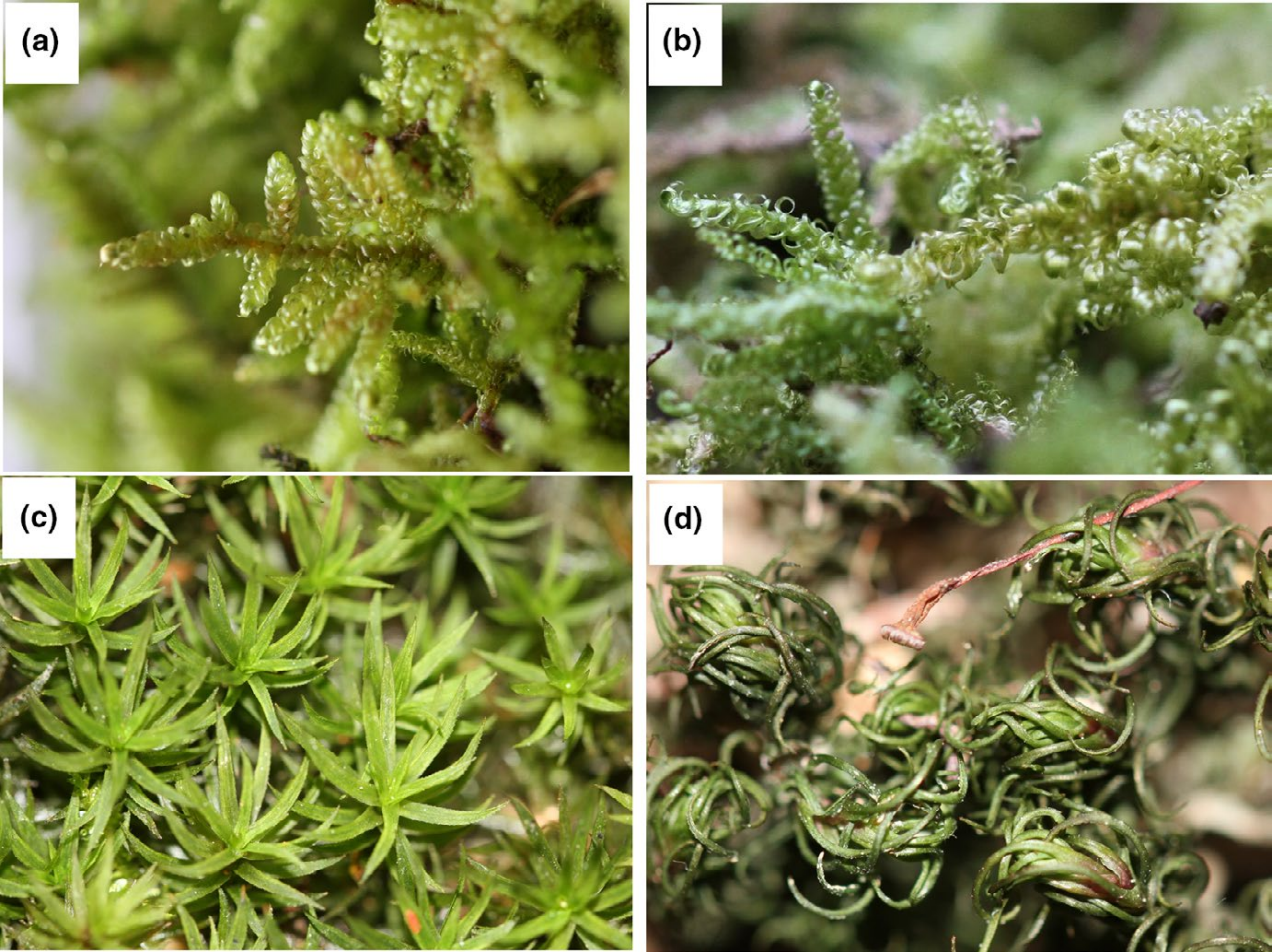
Photographs of (a) controlled *Hypnum plumaeforme*, (b) desiccated *Hypnum plumaeforme*, (c) controlled *Pogonatum cirratum*, and (d) desiccated *Pogonatum cirratum* collected from Heishiding Nature Reserve. Water was supplied every 2 days for the controlled samples and was withheld for 12 days for the desiccated samples

*Hypnum plumaeforme* is a widespread species in China, and *Pogonatum cirratum* is also widely distributed but mainly in tropical and subtropical China. The two mentioned species (Figure 1) were selected as test plants because of their wide distribution and good survival after transplanting, which make them suitable for population‐level differentiation study. In the study presented here, samples of two populations of each species at sites with substantially different environments were collected and transplanted in a subtropical nature reserve in southern China. After 16 months of acclimation, they were subjected to several stress regimes. We then determined and compared their antioxidant enzyme activities and levels of both osmolytes and phytohormones. The following hypotheses were tested as follows: Mosses have stress imprinting mechanisms; antioxidant enzymes, osmolytes, and phytohormones are involved in the mechanisms; and there are significant between‐species and population‐level differences in the mosses' stress tolerance. We also expected to find greater differentiation between the two *H. plumaeformae* populations than between the two *P. cirratum* populations, because the latter are geographically closer to each other.

## 2 METHODS AND MATERIALS

### 2.1 Plants and treatment design

**Figure 2:**
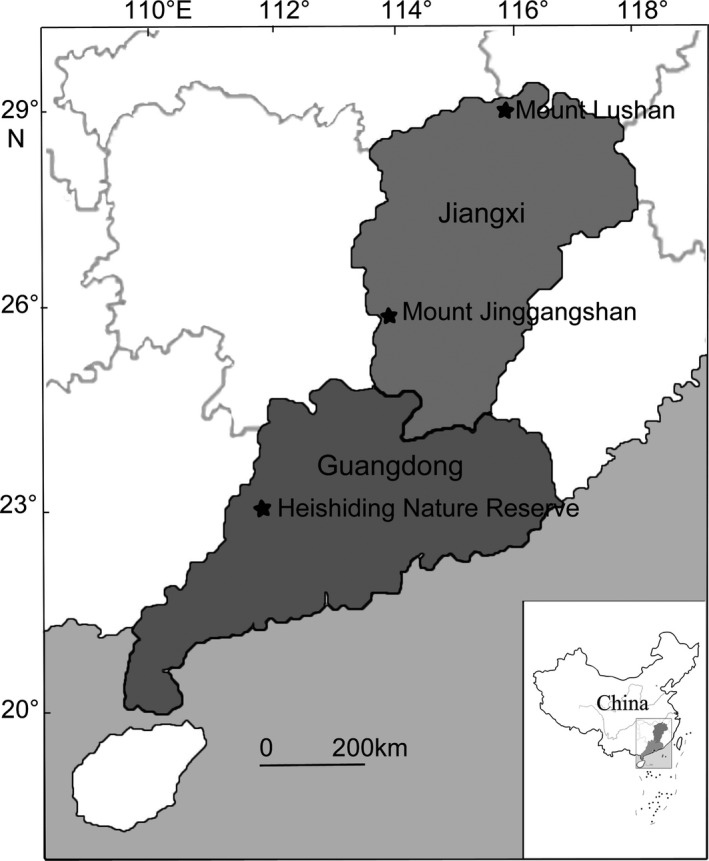
Geographic locations of the sampled populations

**Figure 3:**
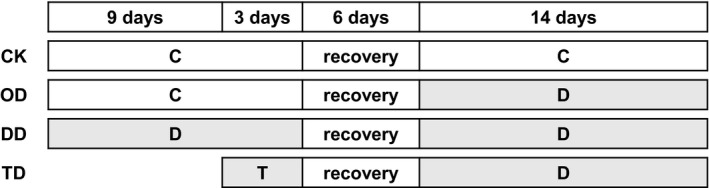
Overview of the treatment regimes. In D treatment periods, no water was supplied to the mosses, but during the other treatment periods, water was supplied every 2 days. The mosses were exposed to ambient temperatures (7–26°C) during C, D, and recovery periods. During T treatment periods, the mosses were placed in a climate chamber providing 12‐hr light/12‐hr dark cycles, with temperatures of 3 and 1°C during the light and dark phases, respectively, and 50 μmol m^−2^ s^−1^ illumination during the light phases

In October 2015, we collected samples of *H. plumaeforme* representing populations located in Heishiding Nature Reserve (23°25′–23°29′N, 111°49′–111°55′E; 400–500 m a.s.l.) and on Mount Lushan (29°26′–29°41′N, 115°52′–116°8′E; 1,000–1,200 m a.s.l.), as well as samples of *P. cirratum* representing populations located in Heishiding Nature Reserve (400–500 m a.s.l.) and on Mount Jinggangshan (26°13′–26°53′N, 113°56′–114°18′E; 800–900 m a.s.l.). The distance between the pairs of *H. plumaeforme* and *P. cirratum* populations was about 800 and 400 km, respectively (Figure 2). Collected mosses were transplanted to a valley bottom at the edge of the forest in Heishiding Nature Reserve as soon as possible. Environmental details of this site have been previously published (Liu, Lei, Jin, et al., 2015; Liu, Liu, Lei, Zhang, & Guo, 2011). Gametophytes of each population were planted in 12 trays (30 × 50 cm) assigned (in triplicate) to four treatment regimes: control (CK), one‐time desiccation stress (OD), duplicated desiccation stress (DD), and cross‐stress (low temperature followed by desiccation stress, TD). The planting density was similar to that of their natural communities. The treatments (each including a 6‐day recovery period between the C, D, or T phase) are graphically illustrated and explained in Figure 3. The mosses were acclimated in the field until February 2017, during which they were watered every 3 days. According to the records of a weather station located in the nature reserve, the mean annual temperature in 2016 is 19.5°C, with an average temperature of the hottest month (August) and coldest month (February) of 26.3°C and 10.3°C, respectively. The annual rainfall in the study area is 2096 mm, with the highest monthly rainfall in August (355 mm) and lowest in December (2.3 mm). In February 2017, trays assigned to the CK, OD, and DD treatments were moved to a wire cage near the valley and watered according to the schedule shown in Figure 3. Six days after the start of these treatments, the TD trays were moved to a climate chamber with the temperature set to 1 and 3°C in 12‐hr light (50 μmol m^−2^ s^−1^) and 12‐hr dark periods, respectively, and the relative humidity (RH) set to 85%, and 3 days later, they were also moved to the wire cage and exposed to the same conditions as the OD samples (Figure 3). During the treatment period (from February 27 to March 30), the mean temperature is 16.2°C and the total rainfall is 121.2 mm, with the RH in the wire cage generally higher than 80% as aperiodically measured using a hygrothermograph (TES 1361, Taiwan). After the first desiccation treatment, both species showed significant morphological difference with the control group (Figure 1) and the water content in desiccated and controlled samples was 0.24 ± 0.03 g/g DW and 1.34 ± 0.24 g/g DW for *P. cirratum*, and 0.27 ± 0.04 g/g DW and 2.42 ± 0.32 g/g DW for *H. plumaeforme*.

Samples of the top 2 cm of gametophytes (ca. 2 g) representing each triplicated species, population, and treatment combination were collected for determination of phytohormones and other physiological indices immediately after the CK, OD, DD, and TD treatments. Samples of *H. plumaeforme* assigned to DD and TD treatments were also collected to determine their phytohormone levels immediately after the 6‐day recovery period. Samples were washed with distilled water for three times to remove debris and sand, and then excess water was blotted with paper sheets. Samples for phytohormone analyses were stored at −80°C prior to determination. Other samples were stored at −20°C and analyzed within 48 hr.

### 2.2 Malondialdehyde and soluble protein contents and activities of superoxide dismutase (E.C. 1.15.1.1), guaiacol peroxidase (EC 1.11.1.7), and catalase (EC 1.11.1.6)

About 0.5 g of each moss sample was weighed and ground with 7 ml ice‐cold 0.1 M phosphate‐buffered saline (PBS) buffer (pH 7.8) containing 0.1 mM ethylenediaminetetraacetic acid‐Na_2_, 3% (m/v) polyvinylpolypyrrolidone, 0.06% (v/v) Triton X‐100, and 5% (v/v) glycerol. The mixture was centrifuged at 12,000 rpm for 5 min at 4°C, and the supernatant was used for measurements.

Malondialdehyde (MDA) content was determined according to the handbook of the Shanghai Institute of Plant Physiology, CAS (Shanghai Institute of Plant Physiology Chinese Academy of Sciences (SIPP), 1999). Superoxide dismutase (SOD) activity was determined by measuring inhibition of the photochemical reduction of nitro blue tetrazolium (NBT; Yu & Rengel, 1999), as previously described (Liu, Lei, Shu, et al., 2015). Catalase (CAT) activity was determined spectrophotometrically by monitoring decomposition of H_2_O_2_ at 240 nm, as follows (Beers & Sizer, 1952). A 0.2 ml portion of each extract was added to 2.5 ml of 0.1 M PBS (pH 7.8). The reaction was initiated by adding 0.5 ml of 0.1 M H_2_O_2_, and 1 unit of CAT activity was defined as the amount of enzyme causing a 0.1 per min decline in OD_240_. Total guaiacol peroxidase (POX) activity was determined by monitoring formation of tetraguaiacol from guaiacol at 470 nm for 4 min in the presence of H_2_O_2_ (Shanghai Institute of Plant Physiology Chinese Academy of Sciences (SIPP), 1999). In these assays, portions (0.4 ml) of the extracts were added to 30 ml of 0.1 M PBS (pH 6.0) and 1.4 µl guaiacol. The reaction was initiated by adding 0.85 µl of 30% H_2_O_2_, and 1 unit of POX activity was defined as the amount of enzyme causing a 0.01 per min reduction in OD_470_. In both CAT and POX assays, portions of boiled extract served as blanks.

### 2.3 Proline contents

Proline was extracted using sulfosalicylic acid, as previously described (Wang & Huang, 2015). About 0.5 g of each sample was ground with 5 ml extraction solution (3% sulfosalicylic acid) and transferred to a test tube, then boiled for 10 min. After cooling to room temperature and centrifugation at 6,000 rpm for 10 min, the supernatant was used to determine proline contents following Troll and Lindsley (1955), with minor modification. Briefly, a 2 ml portion of each extract was mixed with 2 ml glacial acetic acid and 2 ml of 2% ninhydrin in ethanol‐glacial acetic‐phosphoric acid solution (25:60:15, by volume) then boiled for 40 min. After cooling, 3.5 ml of toluene was added, and the mixture was shaken vigorously and left to stand until it had delaminated. The toluene phase was collected and added to 0.5 ml methanol then analyzed spectrophotometrically at 520 nm. Toluene was used for blanks.

### 2.4 Soluble sugar contents

The samples' soluble sugar contents were determined according to SIPP (Shanghai Institute of Plant Physiology Chinese Academy of Sciences (SIPP), 1999). Samples of about 0.3 g were weighed and ground in liquid nitrogen, then 20 ml of ice‐cold 80% ethanol was added, and the samples were ground again for 3–5 min. Each mixture was transferred to a conical flask, extracted at 80°C for 30 min, and then (after cooling) subjected to negative‐pressure filtration. The filtrate was diluted to 50 ml and transferred to a new conical flask, 0.5 ml of saturated lead subacetate solution was added, and the mixture was shaken vigorously. Then, 0.2 g sodium oxalate was added, and the mixture was refiltered. A portion (0.5 ml) of the resulting filtrate was added to 0.5 ml 80% ethanol and 5 ml anthrone reagent (containing 0.2% anthrone and 76% H_2_SO_4_), boiled for 2 min, and after cooling analyzed spectrophotometrically at 630 nm. Sucrose was used as a calibration standard.

### 2.5 Phytohormone contents

ABA, JA, SA, *cis*‐zeatin (*cis*‐Z), *cis*‐zeatin riboside (*cis*‐ZR), *trans*‐zeatin (*trans*‐Z), and IAA contents were extracted, purified, and analyzed by solid phase extraction followed by ultraperformance liquid chromatography coupled to electrospray‐tandem mass spectrometry (UPLC/ESI‐MS/MS), as previously described (Cao, Ma, Mou, Yu, & Chen, 2015; Liu, Li, Xiao, & Wang, 2012).

Samples (0.2 g FW) were added to 1.5 ml extraction solvent (methanol:water:methane acid, 7.9:2:0.1, v/v/v) in 5‐ml microcentrifuge tubes with two steel balls (D = 4 cm) in each tube and broken using a Tissuelyser‐48 multisample tissue grinder (Jingxing, Shanghai) for 3 min. Then, the samples were extracted by ultrasonication (40 kHz, 30 min) on ice, centrifuged (4,000 rpm, 3 min, at 4°C), and extracted at 4°C for 12 hr. After centrifugation (12,000 rpm for 20 min at 4°C), the supernatant was collected and the pellet was reextracted with 1.0 ml of extraction solvent. Then, supernatants were combined and purified using a MAX SPE column (60 mg, 3 ml, Waters, USA) conditioned with 4 ml methanol followed by 2 ml of 0.1 M NH_4_NO_3_. For this, each sample was added to the column, which was then washed with 2 ml of 0.1 M NH_4_NO_3_ followed by 2 ml of 60% methanol containing 0.1 M NH_4_NO_3_. Analytes were eluted with 2.5 ml of 70% methanol containing 1.25 M methane acid. The eluate was dried completely using a vacuum concentrator and redissolved in 0.15 ml of initial mobile phase (acetonitrile:methane acid:water, 5:0.1:94.9, v/v/v) and filtered through a 0.20 μm Nylon filter (Lumeng).

Samples (3 μl) were then analyzed by ultraperformance liquid chromatography–electrospray‐tandem mass spectrometry (UPLC/ESI‐MS/MS). Levels of phytohormones in the samples were quantified using calibration curves obtained from analyses of external standard solutions of IAA, *cis*‐zeatin (*cis*‐Z), *cis*‐zeatin riboside (*cis*‐ZR), *trans*‐zeatin (*trans*‐Z), ABA, JA, and SA (Sigma‐Aldrich). Presented results indicate averages obtained from three injections of each sample. The UPLC system consisted of an Acquity UPLC™ System (Waters) quaternary pump coupled to an autosampler and a Waters Acquity UPLC HSS T3 column (100 × 2.1 mm, 1.7 μm). The mobile phase consisted of a gradient of A:B, time (min) as (95:5, 1), (30:70, 1), and (95:5, 1), where A is 0.1% methane acid in water and B is 0.1% methane acid in acetonitrile, at a constant flow rate of 0.4 ml/min. A XevoTM TQ‐S Tandem Quadrupole mass spectrometer (Waters MS Technologies, Milford, MA, USA) was used for the MS and MS/MS analyses in MRM (multiple reaction monitoring) mode. For analyses of IAA and cytokinins (including *cis*‐Z, *cis*‐ZR, and *trans*‐Z), we used a Turbo Ionspray source in positive ion mode, with 2.5 kV capillary voltage for IAA and 3.0 kV for cytokinins; 22 V cone voltage for IAA and 10 V for cytokinins; 22 V collision voltage for IAA, *cis*‐Z, and *trans*‐Z; and 20 V for *cis*‐ZR. Quantitative data were obtained using peaks of 176.0, 220.0, 220.0, and 352.1 m/z precursor ions together with peaks of 130.3, 118.8, 135.9, and 220.1 m/z production ions for IAA, *cis*‐Z, *trans*‐Z, and *cis*‐ZR, respectively. ABA, JA, and SA were analyzed using the Turbo Ionspray source in negative ion mode, with 1.5 kV capillary voltage, 18 V cone voltage, and collision voltage set at 30, 25, and 20 V for ABA, JA, and SA, respectively. Quantitative data were obtained using peaks of 263.0, 209.0, and 136.8 m/z precursor ions together with peaks of 152.9, 58.8, and 93.0 m/z production ions, respectively.

### 2.6 Data analysis

Means and standard errors of each measured variable were calculated. Differences between treatments within each population were analyzed using one‐way ANOVA, and Tukey's test (or Dunnett's test when homogeneity of variance requirements was not met) was used to identify differences significant at the 0.05 probability level. Log‐transformation was applied when necessary. In addition, between‐treatment and between‐population differences were explored by two‐way ANOVA. Differences in variables are regarded as significant if *p* < 0.05, and only mentioned if they meet this criterion. Increases or decreases in variables refer to measured differences between stressed samples and corresponding controls after the treatments unless otherwise stated.

## 3 RESULTS

### 3.1 Oxidative stress

**Figure 4:**
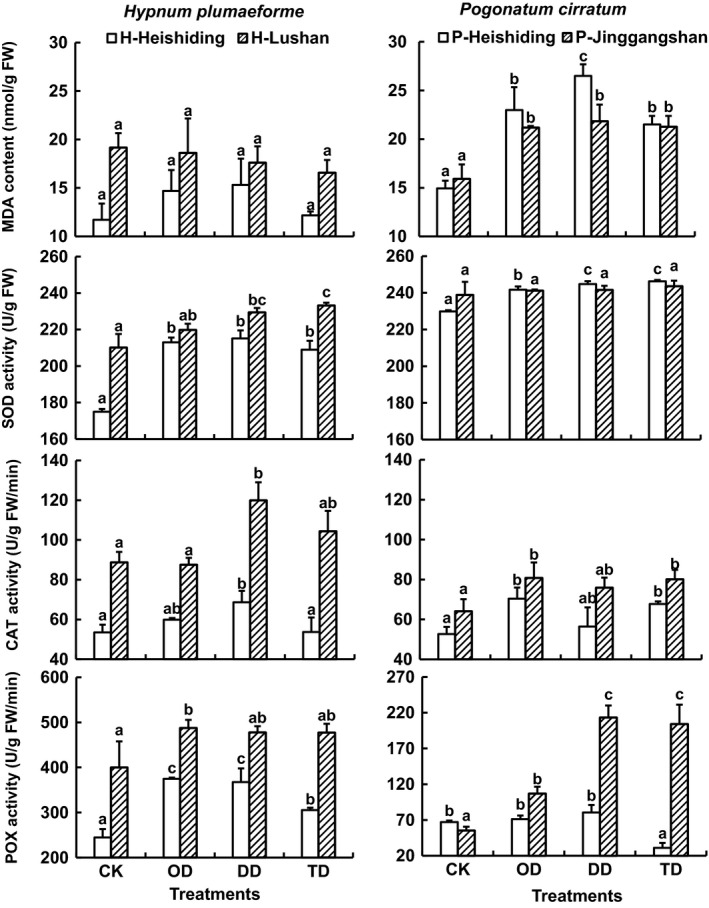
Malondialdehyde (MDA) contents and activities of superoxide dismutase (SOD), catalase (CAT), and guaiacol peroxidase (POX) in *Hypnum plumaeforme* and *Pogonatum cirratum* gametophytes after indicated stress treatments (mean + 1 *SE*, *n* = 3). CK, OD, DD, and TD refer to control, one‐time desiccation stress, duplicated desiccation stress, and low temperature followed by desiccation stress, respectively. Different lowercase letters above columns denote significant difference between treatments within each population (one‐way ANOVA with Tukey's post hoc test). H‐Heishiding and H‐Lushan refer to Heishiding and Lushan populations of *H. plumaeforme*, respectively, while P‐Heishiding and P‐Jinggangshan refer to Heishiding and Jinggangshan populations of *P. cirratum*, respectively. Note the difference in scales for POX activities in the two moss species

The stress treatments stimulated accumulation of antioxidant enzymes but did not cause significant changes in MDA contents of *H. plumaeforme*. In the Heishiding population of *H. plumaeforme* (hereafter, H‐Heishiding), SOD and POX activities increased under all stress treatments, but CAT activity only under the DD treatment. In samples of the Lushan population of *H. plumaeforme* (H‐Lushan), SOD activities increased under DD and TD treatments, while CAT and POX activities increased under DD and OD treatments, respectively (Figure 4). Results of two‐way ANOVA showed that MDA contents and activities of all assayed antioxidant enzymes were higher in H‐Lushan samples than in H‐Heishiding samples (Figure 4, Table 1).

**Table 1:** Effects of population (H‐Heishiding and H‐Lushan) and treatment (control, one‐time desiccation stress, duplicated desiccation stress, and cross‐stress) on oxidative and osmotic indices in *Hypnum plumaeforme* as shown by *F*‐values and *p*‐values (*p*‐values in parentheses) from two‐way ANOVA

	*df*	MDA	SOD	CAT	POX	Pro	SS	SP
Population (P)	1	28.5 (<0.001)	150.5 (<0.001)	246.1 (<0.001)	162.6 (<0.001)	84.0 (<0.001)	83.8 (<0.001)	209.8 (<0.001)
Treatment (T)	3	**1.55 (0.241)**	72.3 (<0.001)	15.6 (<0.001)	21.0 (<0.001)	65.6 (<0.001)	114.1 (<0.001)	54.7 (<0.001)
T × P	3	**1.61 (0.227)**	14.1 (<0.001)	4.96 (0.013)	**2.1 (0.147)**	10.5 (<0.001)	8.4 (0.001)	21.3 (0.001)

Non‐significant effects with *p* > 0.05 are highlighted in bold.

MDA contents were significantly higher in stressed *P. cirratum* samples than in the controls, and the DD treatment caused greater increases in samples of the Heishiding population of *P. cirratum* (P‐Heishiding) than the OD and TD treatments (Figure 4). SOD activities in samples of P‐Heishiding also increased under stress conditions, and both the DD and TD treatments caused stronger increases than the OD treatment. However, the stress treatments did not significantly affect SOD activity in the Jinggangshan population of *P. cirratum* (P‐Jinggangshan). OD and TD (but not DD) treatments caused significant increases in CAT activities in both populations. POX activities in P‐Jinggangshan samples were significantly increased by the OD treatment, and the DD and TD treatments led to further increases, but in P‐Heishiding samples, the TD treatment caused a decrease in POX levels (Figure 4). Differences in SOD activities between the two *P. cirratum* populations were not significant, but CAT and POX activities were significantly higher in P‐Jinggangshan samples than in P‐Heishiding samples (Figure 4, Table 2).

**Table 2:** Effects of population (P‐Heishiding and P‐Jinggangshan) and treatment (control, one‐time desiccation stress, duplicated desiccation stress, and cross‐stress) on oxidative and osmotic indices in *Pogonatum cirratum* as shown by *F*‐values and *p*‐values (*p*‐values in parentheses) from two‐way ANOVA

	*df*	MDA	SOD	CAT	POX	Pro	SS	SP
Population (P)	1	6.7 (0.020)	**0.26 (0.618)**	30.6 (<0.001)	245.9 (0.001)	406.8 (<0.001)	**1.5 (0.236)**	23.6 (<0.001)
Treatment (T)	3	45.8 (<0.001)	14.1 (<0.001)	10.6 (<0.001)	49.1 (<0.001)	62.1 (<0.001)	261.2 (<0.001)	46.6 (<0.001)
T × P	3	4.9 (0.014)	5.3 (0.010**)**	**0.728 (0.550)**	65.7 (<0.001)	6.5 (0.004)	**2.4 (0.105)**	12.2 (<0.001)

Non‐significant effects with *p* > 0.05 are highlighted in bold.

### 3.2 Osmolytes

**Figure 5:**
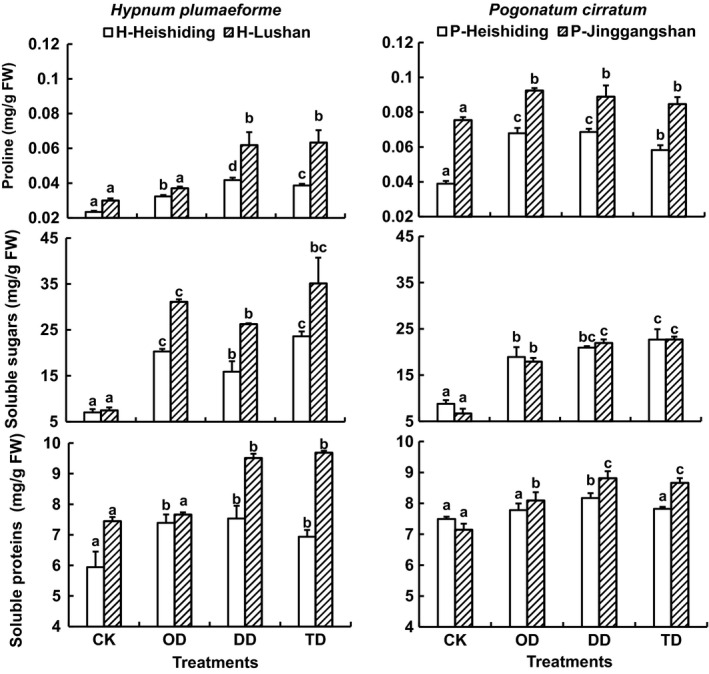
Proline, soluble sugar, and soluble protein contents in gametophytes of *Hypnum plumaeforme* and *Pogonatum cirratum* subjected to indicated stress treatments (mean + 1 *SE*, *n* = 3). CK, OD, DD, and TD refer to control, one‐time desiccation stress, duplicated desiccation stress, and low temperature followed by desiccation stress, respectively. Different lowercase letters above columns denote significant difference between treatments within each population (one‐way ANOVA with Tukey's post hoc test). H‐Heishiding and H‐Lushan refer to Heishiding and Lushan populations of *H. plumaeforme*, respectively, while P‐Heishiding and P‐Jinggangshan refer to Heishiding and Jinggangshan populations of *P. cirratum*, respectively

The stress treatments caused significant accumulation of proline, soluble sugars, and soluble proteins in both mosses. In *H. plumaeforme*, DD and TD treatments caused higher accumulation of proline than OD, while in *P. cirratum*, contents of soluble sugars and soluble proteins were higher in DD and TD samples (except for soluble proteins in TD samples of the P‐Heishiding population) than in OD samples (Figure 5). Results of two‐way ANOVA showed that contents of proline, soluble sugars, and soluble proteins were generally higher in allochthonous populations than in native populations (except for soluble sugars in *P. cirratum*) (Table 1).

### 3.3 Phytohormones

**Figure 6:**
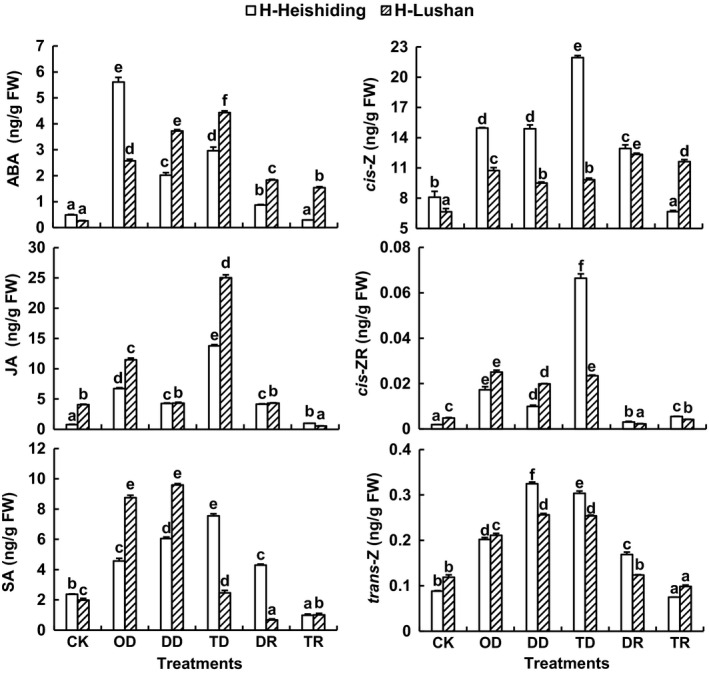
ABA, JA, SA, IAA, *cis*‐Z, *cis*‐ZR, and *trans*‐Z contents in gametophytes of *Hypnum plumaeforme* subjected to indicated stress treatments (mean + 1 *SE*, *n* = 3). CK, OD, DD, and TD refer to control, one‐time desiccation stress, duplicated desiccation stress, and low temperature followed by desiccation stress. DR and TR indicate contents measured after 6 days of recovery from the first desiccation or low temperature treatment. Different lowercase letters above columns denote significant difference between treatments within each population (one‐way ANOVA with Tukey's post hoc test). Data were log‐transformed before further analysis

The stresses caused sharp increases in ABA contents in *H. plumaeforme*: up to 11.4‐ and 16.7‐fold in H‐Heishiding and H‐Lushan samples, respectively (Figure 6). The DD and TD treatments caused higher rises than the OD treatment in H‐Lushan samples, but the OD treatment induced the highest levels in H‐Heishiding samples. JA and SA levels also increased under the stresses. In samples of both *H. plumaeforme* populations, the DD treatment caused lower increases in JA contents than the OD and TD treatments. However, changes in SA contents of the two populations differed. The OD and DD treatments resulted in greater increases than the TD treatment in H‐Lushan samples, but the TD treatment caused the greatest increase in H‐Heishiding samples. After the recovery (R) period, ABA, JA, and SA contents were all lower in samples that had been subjected to D or T stresses (designated DR and TR, respectively) than in OD, DD, and TD samples. However, ABA contents of DR samples and both JA and SA contents of DR samples of the H‐Heishiding population were still significantly higher than those of corresponding controls. In addition, ABA, JA, and SA contents were higher in DR samples than in TR samples of both populations, but the differences were strongest in H‐Heishiding samples (Figure 6).

**Figure 7:**
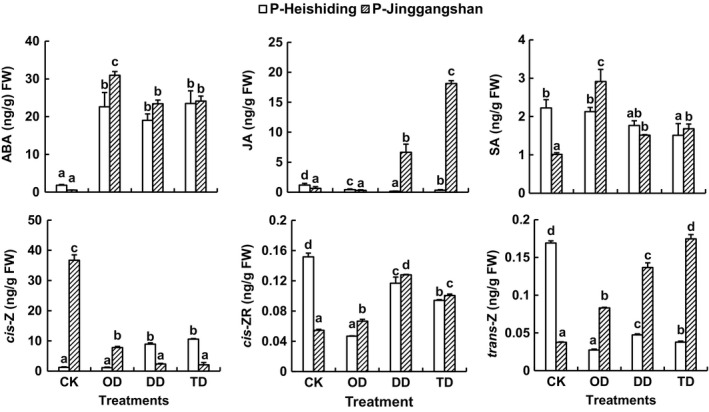
ABA, JA, SA, IAA, *cis*‐Z, *cis*‐ZR, and *trans*‐Z contents in gametophytes of *Pogonatum cirratum* subjected to indicated stress treatments (mean + 1 *SE*, *n* = 3). CK, OD, DD, and TD refer to control, one‐time desiccation stress, duplicated desiccation stress, and low temperature followed by desiccation stress. Different lowercase letters above columns denote significant differences between treatments within each population (one‐way ANOVA with Tukey's post hoc test, in all cases except JA in P‐Jinggangshan, for which Dunnett's test was applied). Data were log‐transformed before further analysis

Sharp increases in ABA content after the stress treatments were also observed in *P. cirratum* samples: up to 12.8‐ and 57.8‐fold in P‐Heishiding and P‐Jinggangshan samples, respectively (Figure 7). Moreover, the duplicated and cross‐stress treatments caused further increases in JA contents of P‐Jinggangshan samples, while SA levels were highest in one‐time stressed samples (Figure 7). In sharp contrast, the stresses did not increase (and in some cases decreased) JA and SA contents in P‐Heishiding samples.

Contents of the cytokinins *cis*‐Z, *trans*‐Z, and *cis*‐ZR widely varied, with ranges of 6.7–21.9, 0.07–0.31, and 0.002–0.066 ng/g FW, respectively, in *H. plumaeforme*, and ranges of 1.23–36.72, 0.03–0.17, and 0.05–0.15 ng/g FW, respectively, in *P. cirratum* (Figures 6 and 7).

The stress treatments caused increases in contents of *cis*‐Z, *cis*‐ZR, and *trans*‐Z in *H. plumaeforme*. After the 6‐day recovery period, the *cis*‐ZR and *trans*‐Z contents of DR and TR samples were lower than in OD, DD, and TD samples, but *cis*‐Z levels were only significantly lower in H‐Heishiding samples, in which levels of *cis*‐Z and *trans*‐Z were higher in DR samples than in TR samples (Figure 6).

The patterns of cytokinin responses showed substantial population‐level differences in *P. cirratum*. In P‐Heishiding samples, the stresses induced increases in *cis*‐Z levels and reductions in *cis*‐ZR and *trans*‐Z levels, while almost opposite changes were observed in P‐Jinggangshan samples (Figure 7).

**Figure 8:**
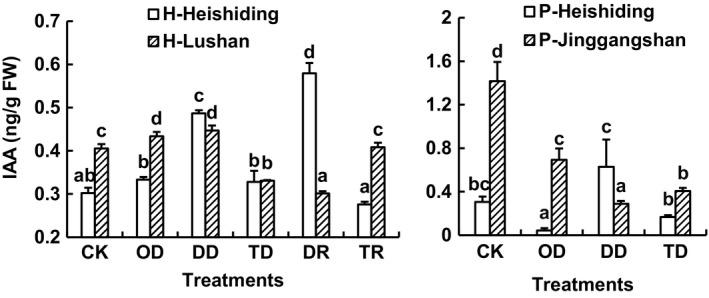
Mean (+1 *SE*, *n* = 3) IAA contents in gametophytes of *Hypnum plumaeforme* and *Pogonatum cirratum* subjected to indicated stress treatments. CK, OD, DD, and TD refer to control, one‐time desiccation stress, duplicated desiccation stress, and low temperature followed by desiccation stress. DR and TR indicate contents measured after 6 days of recovery from the first desiccation or low temperature treatment. Different lowercase letters above columns denote significant difference between treatments within each population (one‐way ANOVA with Tukey's post hoc test). Data were log‐transformed before further analysis. H‐Heishiding and H‐Lushan refer to Heishiding and Lushan populations of *H. plumaeforme*, respectively, while P‐Heishiding and P‐Jinggangshan refer to Heishiding and Jinggangshan populations of *P. cirratum*, respectively

There were no clear patterns in responses of IAA to the stresses in either species (Figure 8).

## 4 DISCUSSION

### 4.1 Antioxidant enzyme activities and osmotic regulation

Stress conditions typically result in overproduction of reactive oxygen species (ROS) and lipid peroxidation in plants (Deeba et al., 2012). Thus, an indicator of lipid peroxidation, MDA, is also often used as an indicator of oxidative stress in plants. Although there has been reported that the membrane damage of *Fontinalis antipyretica* by dehydration is due to nitric oxide end products rather than MDA (Cruz de Carvalho, Catalá, Branquinho, Marques da Silva, & Barreno, 2017), we have found significant increase in MDA in *H. plumaeforme* and *P. cirratum* upon exposure to water stress (Liu, Lei, Jin, et al., 2015). On the other hand, stress conditions generally induce increases in activities of antioxidant, ROS‐scavenging enzymes such as SOD, CAT, and POX (Sofo, Dichio, Xiloyannis, & Masia, 2004). Our results showed that stresses stimulated increases in activities of SOD, CAT, and POX in both species, but significant increases in MDA levels were only observed in *P. cirratum*. These observations suggest that the stresses triggered antioxidant defense mechanisms that neutralized ROS sufficiently to avoid significant cell membrane damage in *H. plumaeforme*, but *P. cirratum* is more sensitive to water stress and the treatments caused damage to its membranes. Proline, soluble sugar, and soluble proteins are osmolytes, which often accumulate in plants upon exposure to osmotic stress (Ahmad et al., 2016; Cruz de Carvalho, Bernades da Silva, Branquinho, & Marques da Silva, 2015; Cruz de Carvalho et al., 2014; Mittal, Kumari, & Sharma, 2012; Nagao et al., 2005), and our results show that the stresses also led to their accumulation in the mosses.

In previous studies, we found that after a 10‐day recovery period stressed mosses still maintained relatively high levels of SOD and osmolytes (Liu et al., 2016; Liu, Lei, Jin, et al., 2015). In the present study, we found that proline, soluble sugar, and soluble protein contents were higher after duplicated and cross‐stress treatments than after one‐time stress treatments. These results indicate that the first stress primed accumulation of osmolytes in the mosses when subjected to the second stress, and improved their adaptation to the later stress. Thus, they confirm two of our hypotheses: that mosses have stress imprinting mechanisms, and osmolytes are involved in them. These results also indicated that the stress imprinting can be maintained in the tested mosses for 6 days. Further increases in SOD and CAT activities in H‐Lushan, SOD in P‐Heishiding, and POX in P‐Jinggangshan samples subjected to duplicated or cross‐stresses indicated that the first stress also induced stronger increases in ROS‐scavenging activities in the mosses during exposure to the later stress (Ye & Gressel, 2000). On the other hand, POX activities were lower in TD samples of the Heishiding populations of both species, but not the allochthonous populations, than in corresponding OD and DD samples, highlighting the differences in low temperature responses between populations from different latitudes.

Moreover, even after a year of acclimation in Heishiding Nature Reserve, levels and activities of MDA, antioxidant enzymes, and osmolytes were still much higher in H‐Lushan than in H‐Heishiding samples. However, there were no substantial differences in SOD and soluble sugar contents or activities between the native and allochthonous populations of *P. cirratum*. These results indicate that population‐level differentiation is present in both species, but more strongly in *H. plumaeforme* than in *P. cirratum*. This is in accordance with our third hypothesis and suggests that geographical distance might be one of the drivers of population‐level differentiation (Chambers & Emery, 2016; Korpelainen et al., 2005), though further studies are needed to illustrate the role of geological distance in their population differentiation.

### 4.2 Phytohormone regulation

ABA plays key roles in mosses' stress tolerance (Beckett, 1999; Beckett, Csintalan, & Tuba, 2000; Bhyan et al., 2012; Mayaba, Beckett, Csintalan, & Tuba, 2001; Nagao et al., 2005; Xiao, Yobi, Koster, He, & Oliver, 2018). Our results show that stresses induced sharp increases in ABA contents in both species, in accordance with previous studies (Kohli et al., 2013; Xiao et al., 2018). JA and SA are generally considered to be largely involved in biotic defenses (Bari & Jones, 2009), but they also accumulate in plants during abiotic stress and can participate in regulation of abiotic stress responses (Hu, Jiang, Wang, & Yu, 2013; Singh & Usha, 2003; Wang et al., 2010). We found no previous studies on responses of JA and SA to stress in mosses (literature screening procedures not described). However, our study demonstrates that stresses induced increases in JA and SA levels in both populations of *H. plumaeforme*, as observed in vascular plants, but only in P‐Jinggangshan samples of *P. cirratum*.

Cytokinins are a large group of phytohormones, mostly *N* ^6^‐substituted adenine derivatives, which are strongly associated with growth and development, and generally considered to be negative regulators of abiotic stress signaling (Nishiyama et al., 2011; Verslues, 2016). Short‐term or mild stresses generally stimulate accumulation of cytokinins, while prolonged or more severe stresses are generally associated with downregulation of active cytokinin levels (Ha, Vankova, Yamaguchi‐Shinozaki, Shinozaki, & Tran, 2012; Havlová et al., 2008; Nishiyama et al., 2011). However, no studies on responses of cytokinins to stresses in mosses have been reported (literature screening procedures not described). *Cis*‐Z and *trans*‐Z are generally the most abundant cytokinin species in mosses (Drábková, Dobrev, & Motyka, 2015; Gajdošová et al.,^2011^), and our results show that *cis*‐Z was almost 2 orders of magnitude more abundant than *trans*‐Z in the two species considered here. In addition, stresses induced accumulation of *cis*‐Z, *cis*‐ZR, and *trans*‐Z in both populations of *H. plumaeforme*, but in *P. cirratum,* there were differences in responses both among cytokinins and between populations.

After the 6‐day recovery period, the elevated JA, SA, *cis*‐Z, *cis*‐ZR, and *trans*‐Z contents in stressed *H. plumaeforme* samples had decreased significantly toward control levels. Such reductions in levels of stress response substances after recovery, and enhancement of defense mechanisms only when stress recurs, avoid costs of constitutive activation of stress responses (Brinda et al., 2016; Bruce et al., 2007; Cruz de Carvalho et al., 2014). However, their ABA levels were still higher than in control samples after recovery, which might promote faster and better responses to later stresses as most stress responses are ABA‐mediated (Bopp & Werner, 1993; Hellwege et al., 1994; Kohli et al., 2013).

Upon exposure to duplicated or cross‐stresses, we detected further accumulations of *trans*‐Z in samples of both *H. plumaeforme* populations, and of *cis*‐Z in H‐Heishiding samples, which might be associated with stress imprinting. Moreover, we detected substantial between‐hormone, between‐species, and between‐population variations in responses, reflecting the sensitivity and complexity of phytohormone regulation in stress responses. Due to the complex crosstalk among phytohormones (Kohli et al., 2013; Verma et al., 2016) as well as the involvement of epigenetic changes in stress responses, more detailed and specific studies are needed to elucidate the underlying mechanisms of hormone regulation in the mosses' stress imprinting.

## 5 CONCLUSION

Desiccation stress induced increases in antioxidant enzyme activities and contents of osmolytes and ABA in the mosses. Though with substantial between‐species and population‐level differences, the first desiccation or low temperature stress led to further increases in levels or activities of osmolytes and antioxidant enzymes upon exposure to the later desiccation stress after 6 days of recovery, indicating that the stress imprinting in the mosses can be maintained for at least 6 days. Significantly higher levels or activities of MDA, SOD, CAT, POX, and osmolytes were detected in the allochthonous population of *H. plumaeforme* than in the native population even after one year of acclimation, indicating distinct population differentiation, but the differentiation between the populations of the *P. cirratum* was weaker, which might be related to their closer geographical distance.

## CONFLICT OF INTEREST

None declared.

## AUTHOR CONTRIBUTIONS

WQ Liu was responsible for the design and implementation of the entire study. JQ Xu and WQ Liu carried out the study, including fieldwork, laboratory experiments, data analysis, and drafting the manuscript. W Fu, XY Wang, and YF Chen participated in the laboratory experiments, data analysis, and drafting the manuscript. CY Lei collected the weather data during the study period and participated in the fieldwork and laboratory experiments. WQ Liu, YF Chen, and CY Lei critically revise the manuscript. All authors gave the final approval of the version to be published.

## DATA ACCESSIBILITY

The data of ROS, osmolytes, and phytohormones: Dryad https://doi.org/10.5061/dryad.833qr19.
